# Urinary volatilomics for biomarker discovery in kidney stone patients and healthy controls

**DOI:** 10.1007/s00240-026-02045-7

**Published:** 2026-08-25

**Authors:** Pascal Fuchsmann, Eugenia Pertziger, Hélène Y. Meng, Saša M. Miladinović, Murielle Bochud, Carsten A. Wagner, Olivier Bonny, Guy Vergères

**Affiliations:** 1 Agroscope, Food Microbial Systems Research Division, Bern, Switzerland; 2 Institute of Life Sciences, School of Engineering, University of Applied Sciences West Switzerland, Sion, Switzerland; 3 Department of Epidemiology and Health Systems, Center for Primary Care and Public Health (Unisanté), University of Lausanne, Lausanne, Switzerland; 4 Institute of Physiology, University of Zurich, Zurich, Switzerland; 5 Department of Biomedical Sciences, University of Lausanne, Lausanne, Switzerland; 6 Service of Nephrology and Hypertension, Lausanne University Hospital, Lausanne, Switzerland; 7 Service of Nephrology, Fribourg State Hospital, Fribourg, Switzerland

**Keywords:** Nephrolithiasis, Urinary volatile organic compounds (VOCs), Metabolomics, GC–MS profiling

## Abstract

Nephrolithiasis is a prevalent and recurrent condition with a rising incidence worldwide, largely attributed to modifiable lifestyle factors, including diet. Novel, non-invasive biomarkers are needed to improve early diagnosis, recurrence monitoring, and personalized prevention. This study aimed to identify urinary volatile organic compounds (VOCs) that differentiate healthy individuals from kidney stone formers and to explore their associations with fermented food intake. Using dynamic headspace Vacuum in-tube extraction gas chromatography–mass spectrometry (DHS-V-ITEX-GC-MS), 24-h urine samples were analyzed to identify potential volatile biomarkers related to kidney stone risk. This case–control study included 232 patients with recurrent kidney stone disease and 168 healthy controls enrolled in the Swiss Kidney Stone Cohort. Urine samples were analyzed using untargeted volatilomic profiling. Data were normalized, quality-filtered, and adjusted for relevant confounders, including study center, sex, age, body mass index, smoking status, and diabetes status. Uni- and multivariate statistical analyses were applied to identify discriminative features, followed by metabolite identification. Seventy-seven metabolites differed significantly between kidney stone formers and controls. Octane, 1,1′-oxybis- and 6-methyl-5-hepten-2-one were present at higher levels in controls, whereas acetic acid and propanoic acid were detected at higher levels in participants with kidney stones. Among these metabolites, three were associated with fermented food consumption, with 3-nonen-2-one emerging as a potential mediator of the association between coffee consumption and reduced kidney stone risk.

**Supplementary Information:**

The online version contains supplementary material available at 10.1007/s00240-026-02045-7.

## Introduction

Kidney stones are a frequent and recurrent condition affecting 10%–15% of individuals in developed countries during their lifetime, with incidence rising in recent decades [1]. This trend has been attributed to modifiable behaviors such as diet, sedentary lifestyle, and hydration patterns, although factors like obesity and climate exert additional, multifactorial influences [2, 3]. Stones form when urinary solutes exceed solubility limits, promoting crystal nucleation and aggregation. Calcium oxalate stones are most common [4], and nephrolithiasis can progress to chronic kidney disease in high-risk individuals [5], contributing substantially to healthcare burden [6]. Genetic and metabolic predispositions do not fully explain these trends, underscoring the importance of lifestyle and environmental factors [7].

Diet modulates urinary pH, citrate, and lithogenic substrates such as oxalate, calcium and uric acid. Metabolomic studies show that diet-induced metabolic shifts reshape the urinary metabolome in ways that may promote or prevent stone formation [8]. However, little is known about how specific dietary components affect urinary volatile organic compounds (VOCs) in relation to kidney stone risk.

Fermented foods are of particular interest because they represent a major part of human diet [9] and they influence both gut metabolism and urinary metabolite profile. The types and quantities of fermented foods consumed vary substantially across geographic regions and cultural traditions, with particularly high consumption reported in several East Asian and Eastern European populations. Consequently, dietary associations identified in Swiss adults may not be directly generalizable to other populations. Epidemiological data suggest protective associations with kidney health, with higher intake of live microbes linked to reduced stone risk [10]. Diets supporting gut microbial health, reflected by higher DI-GM (Dietary Index for Gut Microbiota) scores, were inversely associated with stone prevalence, plausibly through improved oxalate handling and urinary citrate excretion [11]. Microbial contributions to oxalate metabolism further support this relationship [12, 13].

VOCs are emerging biomarkers in medicine and nutrition [14–16]. Urine provides a stable matrix enriched in endogenous and exogenous metabolites, enabling reproducible volatilomic profiling [17–21]. Advances in gas chromatography–mass spectrometry–based technologies (GC–MS) have improved sensitivity and throughput [22]. The emerging field of nutrivolatilomics explores VOC responses to diet [23–25].

To date, no study jointly profiled urinary VOCs, fermented-food intake, and nephrolithiasis. Here, we aim to identify urinary VOCs that significantly differ between healthy individuals and kidney stone formers, and to examine whether any of these VOCs are associated with fermented-food consumption. Using an optimized DHS-V-ITEX-GC-MS workflow, we characterize urinary volatile compounds that may be differentially produced in stone formers and healthy controls. Our working hypotheses are that (i) stone formers display a distinct urinary VOC pattern and (ii) some of these VOCs may be linked to fermented-food intake.

## Methods

### Study design and population

The Swiss Kidney Stone Cohort (SKSC) is a nationwide prospective study designed to investigate metabolic, genetic, and environmental determinants of nephrolithiasis [26]. For this metabolomic analysis, we included 400 participants (232 cases and 168 controls) selected based on complete dietary data and urine availability [7]. Standardized 24-h dietary recalls were annotated for fermented foods and live microorganisms using the classification system of Pertziger et al. [9], grouped into six food categories and 35 subgroups (**Supplementary Table ****S1**). Participant characteristics are presented in Table 1.

**Table 1:** Characteristics of the participants with two 24-h dietary recalls at baseline, by case-control status. Baseline characteristics of participants with two 24-h dietary recalls, according to kidney stone case-control status. Values are presented as means (SD) or medians (25th–75th percentiles) for continuous variables and as percentages for categorical variables

Variable	Category	Kidney stone formers	Non-kidney stone formers
Sex (%)	Women	36%	46%
Age (years)	All	47.3 [19, 79]	43.4 [20, 81]
mean [min, max]	Men	48.2 [20, 79]	45.7 [22, 81]
	Women	45.6 [19, 73]	40.6 [20, 62]
BMI (kg/m^2^)	All	26.6 (4.7)	25.2 (4.4)
mean (SD)	Men	26.7 (4.5)	26.0 (3.8)
	Women	26.2 (5.2)	24.2 (4.9)
German speaking (%)		57%	55%
Education level (%)	Low	12%	2%
	Middle	57%	40%
	High	31%	58%
Smoking status	Non-smoker	70.50%	72.40%
Diabetes status	No	91.20%	99.20%
	Diabete type 2	8.80%	0.80%
Study center n (%)	Zürich	94 (40.5%)	92 (54.8%)
	Lausanne	68 (29.3%)	56 (33.3%)
	Basel	5 (2.2%)	0 (0.0%)
	Aarau	10 (4.3%)	4 (2.4%)
	Geneva	29 (12.5%)	16 (9.5%)
	Berne	26 (11.2%)	0 (0.0%)
Coffee consumption			
Daily coffee intake (mL/d)	Mean (SD)	173.1 (85.7)	183.4 (122.8)
	Median (P25, P75)	152.1 (125, 202.5)	159.5 (113.1, 200.0)

Recruitment occurred across six Swiss hospitals (Aarau, Basel, Bern, Geneva, Lausanne, Zurich) with standardized procedures for biospecimen collection; samples were frozen at − 80 °C and stored in the centralized University of Zurich biobank [26].

Inclusion criteria for cases were age ≥ 18 years and ≥ 2 stone episodes; control participants were recruited in Geneva, Lausanne and Zurich and proven to be stone free by abdominal CT-scan imaging [26].

**Fig. 1:**
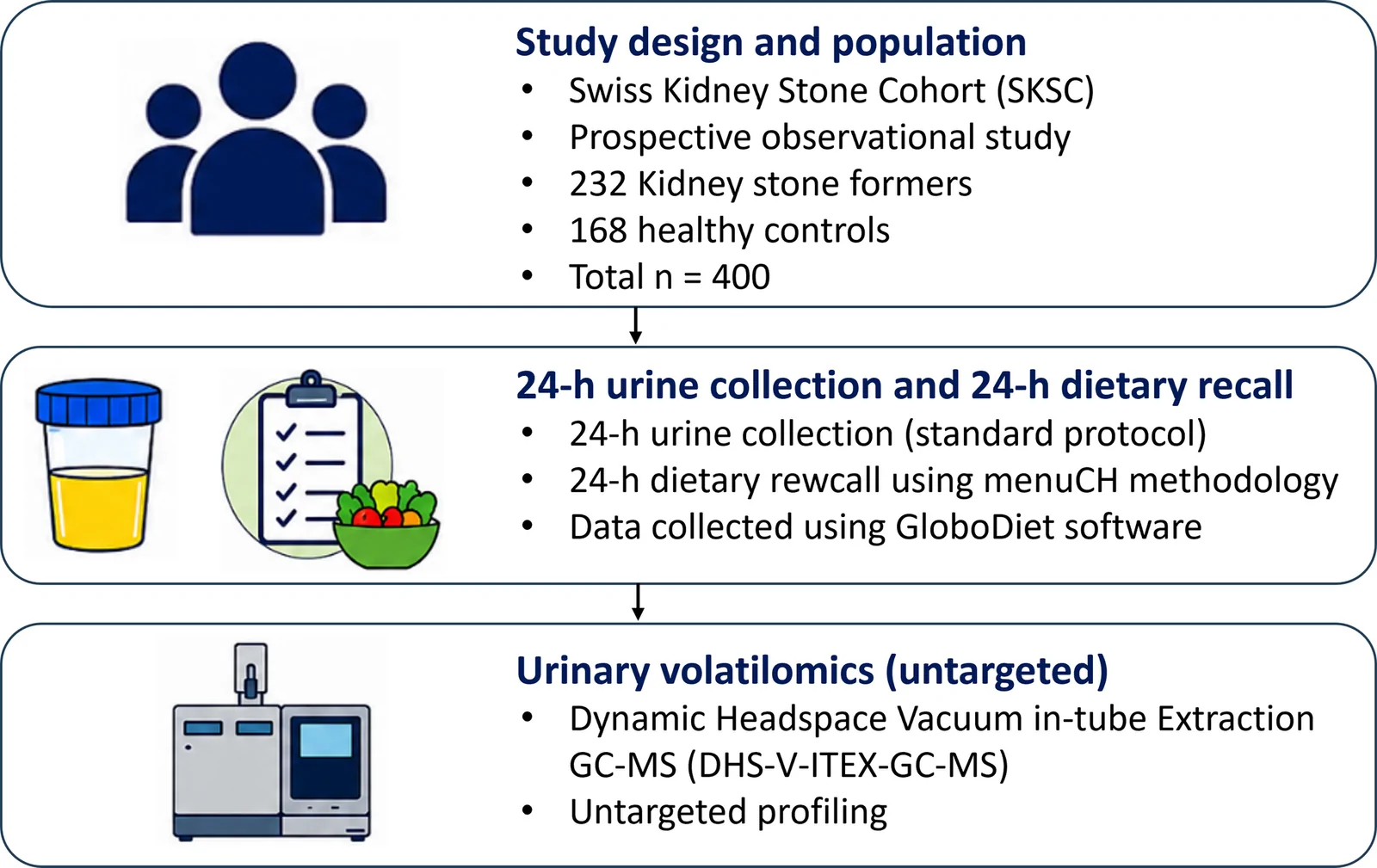
Overview of the study design. Overview of the study design and workflow from participant inclusion to collection of urine, dietary data and untargeted urinary volatilomics

Dietary intake was assessed using standardized 24-h GloboDiet^®^ interviews conducted by trained dietitians and centrally reviewed for quality control [7, 27]. The data collected also included sociodemographic, clinical, lifestyle, and laboratory variables, providing a comprehensive framework for integrative omics analysis. The overall study design and workflow are summarized in Fig. 1.

### Volatilomic data processing and statistical analysis

Urinary volatile organic compounds were analyzed using an untargeted dynamic headspace Vacuum in-tube extraction gas chromatography–mass spectrometry (DHS-V-ITEX-GC-MS) workflow. A standardized analytical workflow was applied to ensure robustness and minimize technical and biological confounding. Detailed analytical conditions and instrumental parameters are provided in the Supplementary Methods. An overview of the data processing and analysis workflow is provided in Supplementary Fig.S1.

Raw GC–MS data were processed using MassHunter Profinder (Agilent). After deconvolution and retention-time alignment, features were filtered using a modified 80% rule to retain robust signals. Data were normalized using probabilistic quotient normalization (PQN) followed by Locally estimated scatterplot smoothing (LOESS) correction based on quality control (QC) injections. Missing values, which occurred only among features passing the modified 80% rule, were imputed using random forest algorithms, and intensities were log_2_-transformed and autoscaled before modelling.

**Fig. 2:**
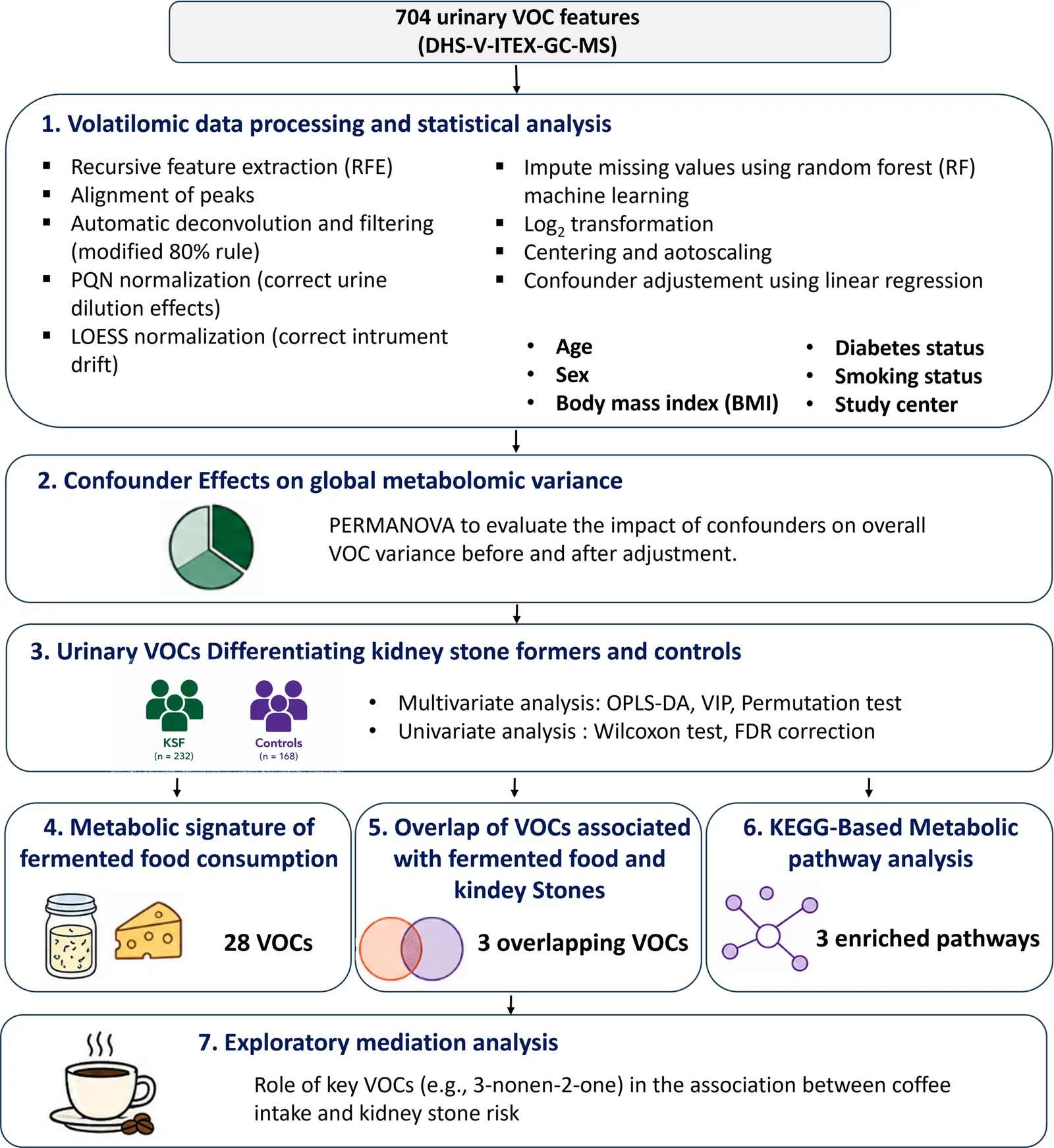
Overview of the statistical analysis workflow. Schematic overview of the statistical analysis pipeline from urinary volatilomics data processing to identification of VOCs associated with kidney stone disease, fermented food consumption and metabolic pathways, and exploratory mediation analysis of the role of key VOCs in the coffee-kidney stone association

To limit confounding, metabolite intensities were adjusted for age, sex, body mass index (BMI), diabetes status, smoking status, and study center using linear regression, based on prior evidence of their influence on urinary metabolomic profiles. The impact of these variables on data structure was explored prior to adjustment (Supplementary Fig. S2). Group comparisons used Wilcoxon rank-sum tests with false discovery rate (FDR) correction. Multivariate analyses using orthogonal projections to latent structures discriminant analysis (OPLS-DA) were performed on covariate-adjusted data to explore metabolic patterns associated with kidney stone status. These analyses highlighted metabolites contributing most strongly to case–control separation, as quantified by variable importance in projection (VIP) scores and evaluated through permutation testing (999 permutations). Metabolite identification followed Metabolomics Standards Initiative (MSI) criteria based on spectral matching and retention indices. An exploratory causal mediation analysis (mediation R package) was performed to evaluate whether selected metabolites may statistically mediate the association between dietary exposures, particularly fermented food intake, and kidney stone status. The complete analytical workflow, from preprocessing of urinary VOCs to exploratory mediation analysis, is summarized in Fig. 2.

### Dietary assessment and definition of fermented food intake

Dietary intake was assessed using interviewer-administered 24-h dietary recalls. Fermented foods were identified according to the definition of the International Scientific Association for Probiotics and Prebiotics (ISAPP) and classified following the methodological framework described by Pertziger et al. in the Swiss National Nutrition Survey menuCH [9]. In this framework, foods were annotated based on documented fermentation processes, Swiss food production standards, and published literature describing microbial composition. Each food item was therefore coded as fermented, non-fermented, or composite with fermented ingredients, and descriptors such as presence of live microorganisms or processing steps (e.g., heat, filtration) were assigned using the same criteria. Detailed descriptive results of fermented food consumption in the Swiss Kidney Stone Cohort have been reported previously [9] and are therefore not repeated here.

Intake of fermented foods was quantified in grams per day based on the dietary recall, mirroring the quantification approach used in menuCH [9]. For statistical analyses, participants were categorized into intake groups (e.g., none, low, or high) based on the distribution of fermented food consumption (median or tertiles).

### Metabolic pathway enrichment analysis

Pathway analysis was performed using metabolites for which a Kyoto Encyclopedia of Genes and Genomes (KEGG) compound identifier (C-number) was available. KEGG identifiers were retrieved from the KEGG database [28]. These KEGG identifiers served as the sole input for pathway inference. The analysis was carried out in R using the FELLA package, which leverages the hierarchical structure and topology of the KEGG knowledge graph (compounds → reactions → enzymes → modules → pathways). FELLA applies a network-diffusion algorithm to propagate the information from the input compounds through the metabolic graph and identify the most relevant upstream biochemical elements and pathways [29].

The resulting diffusion scores were used to determine the pathways and reactions most strongly associated with the identified metabolites. Only pathways highlighted by FELLA as significantly enriched or strongly connected were retained for biological interpretation.

## Results

Untargeted analysis detected 704 urinary VOC features (raw GC–MS signals), with detection frequencies varying across samples, as expected for untargeted profiling. Because these features represent unnamed analytical signals, they are not individually listed; all curated metabolites (80 metabolites) that differentiated kidney stone formers from controls are reported in Supplementary Table S2.

### Confounder Effects on Global Metabolomic Variance

Several demographic and clinical variables contributed significantly to global variance in the metabolomic dataset. Permutational analysis of variance (PERMANOVA) identified study center as the strongest source of variation (R² = 3.55%, *p* = 0.001), followed by sex (R² = 1.37%, *p* = 0.001), while smoking, age, BMI, and diabetes each explained less than 1% of the variance (all *p* < 0.05). These patterns were consistent with the metabolite-wise ANCOVA adjusted for case–control status: sex was associated with 183 metabolites and study center with 161 metabolites (FDR < 0.05), whereas age, BMI, smoking, and diabetes were associated with far fewer features (*n* ≤ 44). Full metrics for each covariate are reported in Table 2, and visualization of confounder-related structure is shown in Supplementary Fig. S2.

**Table 2:** Multimodal assessment of covariate effects on urinary metabolomic profiles (PERMANOVA, ANCOVA, GVIF). The influence of six variables (age, BMI, diabetes, sex, smoking, and study center) was evaluated using ANCOVA, PERMANOVA, and GVIF

Confounder	PERMANOVA		ANCOVA		GVIF
	*R*²	*P*-Value	Median_F	n_sig_FDR	GVIF^(1/(2*Df))^
Study center	0.0355	0.001	1.564	161	1.01
Sex	0.0137	0.001	2.646	183	1.04
Smoking status	0.0075	0.003	0.861	36	1.02
Age	0.0054	0.001	0.811	44	1.09
BMI	0.0039	0.006	1.053	31	1.05
Diabetes status	0.0037	0.016	0.514	21	1.03

R²: explained variance; P: significance level; Median_F: median F-statistic; n_sig_FDR: number of significant features; GVIF^(1/(2×Df))^: adjusted GVIF for comparison across factors

Multicollinearity across covariates was minimal, as indicated by the generalized variance inflation factor (GVIF^(1/(2·df))^ < 1.10 for all variables). These covariates were included as adjustment factors because they were associated with variability in the urinary metabolome and showed associations with either the exposure or the outcome in our dataset, making them relevant candidates for confounding adjustment. Based on these results, metabolite intensities were adjusted for study center, sex, age, BMI, smoking status, and diabetes status before downstream analyses. After adjustment, variance attributable to all covariates decreased markedly, and no variable accounted for more than 0.8% of total variance (R² < 0.008). Study center and sex were no longer statistically significant contributors to global variance, whereas age and BMI retained only small residual effects (R² ≤ 0.006). All subsequent analyses were therefore performed using the adjusted dataset. This adjustment step ensured that downstream group differences primarily reflected disease status rather than residual demographic or center-related effects.

### Urinary VOCs Differentiating Kidney Stone Formers and controls

OPLS-DA of the adjusted urinary VOC dataset (*n* = 400) showed partial separation between kidney stone formers and controls (Supplementary Fig. S3). Compared with unadjusted models, covariate adjustment resulted in reduced model complexity while preserving case–control discrimination. The model displayed good performance (R²X = 0.119, R²Y = 0.705, Q² = 0.507), and permutation testing (1,000 permutations) confirmed that the observed R²Y and Q² values were significantly higher than expected by chance (*p* = 0.001 for both).

Based on VIP scores, 161 features contributed to case–control separation. After filtering to remove poorly detected features and redundant signals corresponding to the same metabolite (Supplementary Table S2), 80 unique metabolites were retained for statistical testing. Among these, 77 features were significantly different between kidney stone formers and controls (FDR < 0.05), with 42 metabolites showing higher abundance in cases and 35 in controls. Chemical annotation yielded 32 metabolites with high-confidence identifications (MSI Level 1 or 2; Table 3).

**Table 3:** Volatile organic compounds (VOCs) identified as potential biomarkers differentiating stone formers from controls. Thirty-two metabolites contributing to the separation between stone formers and controls in the OPLS-DA model are listed. Only compounds with level 1–2 identification confidence were retained. Columns include compound name, CAS number, Human metabolome database (HMDB) ID (if available), KEGG N° (if available), m/z, retention time (RT), calculated and reference retention index (RI) values, and RI from analytical standards. “VIP > 1” denotes variable importance in projection; “Direction” indicates relative abundance (1 = higher in cases, red, − 1 = higher in controls, green). Significance was tested by Wilcoxon rank-sum with FDR correction. The final column reports NIST spectral match scores (%)

Feature_ID	Compound name	CAS *N*°	HMDB *N*°	KEGG *N*°	m/z	Retention time [min]	RI calculated	RI literature	RI standards	Level of identification	VIP_pred_	Direction	wilcoxon *p*-value_FDR	Match
MET_05	Propanal	123-38-6	HMDB0003366	C00479	58	4.02	725	798+-14	780	2	1.73	-1	5.13 × 10^− 06^	83.2
MET_06	Ethanol	64-17-5	HMDB0000108	C00469	46	4.05	727	932+-8	941	2	1.67	-1	1.78 × 10^− 04^	95.3
MET_11	Acetone	64-17-5	HMDB0001659	C00207	43	4.18	734	832	805	2	2.1	-1	3.95 × 10^− 07^	95.3
MET_38	2-pentanone	107-87-9	HMDB0034235	C01949	85	7.99	994	981+-11	988	1	1.2	-1	1.23 × 10^− 02^	91.9
MET_53	Furan, 2,3,5-trimethyl-	10504-04-8	HMDB0029721	ND	110	10.6	1076	1065	ND	2	1.3	1	3.74 × 10^− 03^	85.8
MET_54	2,3-pentanedione	600-14-6	HMDB0031598	ND	43	10.7	1076	1067	ND	1	1.35	1	1.37 × 10^− 03^	95.6
MET_66	(R)-(-)-2-Pentanol	31087-44-2	HMDB0031599	ND	45	12.6	1133	1130	1128	1	1.58	-1	3.18 × 10^− 04^	88.7
MET_146	2-propanol, 1-butoxy-	5131-66-8	ND	ND	45	19.4	1357	1353+-10	1352	1	2.52	1	1.09 × 10^− 13^	87.5
MET_152	5-hepten-2-one, 6-methyl-	110-93-0	HMDB0035915	C07287	73	19.4	1357	1338+-9	1351	1	3.54	-1	6.04 × 10^− 15^	82
MET_175	Dimethyl trisulfide	3658-80-8	HMDB0013780	C08372	79	20.9	1408	1377+-11	1405	1	1.1	-1	4.59 × 10^− 03^	93.6
MET_186	Ethanol, 2-butoxy-	111-76-2	HMDB0031327	C19355	57	21.4	1430	1405+-12	1420	2	2.21	1	3.97 × 10^− 07^	91.8
MET_212	Acetic acid	64-19-7	HMDB0000042	C00033	67	22.7	1479	1449+-13	1472	1	2.84	1	9.32 × 10^− 20^	96.9
MET_217	2-Propanone, 1-(acetyloxy)-	592-20-1	HMDB0034466	ND	43	22.9	1487	1474+-9	1488	1	1.14	1	6.19 × 10^− 04^	94
MET_224	1-hexanol, 2-ethyl-	104-76-7	HMDB0031231	C02498	57	23.3	1502	1491+-5	1499	1	1.62	-1	1.35 × 10^− 02^	97
MET_238	Decanal	112-31-2	HMDB0011623	C12307	57	23.7	1518	1498+-8	1515	1	2.24	-1	1.07 × 10^− 02^	97.8
MET_248	2,5-hexanedione	110-13-4	HMDB0245506	ND	43	24.1	1533	1500+-4	1537	1	1.8	-1	5.27 × 10^− 03^	82.2
MET_252	3-nonen-2-one	14309-57-0	HMDB0034764	ND	55	24.2	1537	1515+-12	1539	1	2.53	-1	9.85 × 10^− 07^	82.2
MET_266	Propanoic acid	79-09-4	HMDB0000237	C00163	74	24.8	1561	1535+-11	1570	1	1.72	1	3.73 × 10^− 04^	96.2
MET_368	Levomenthol	2216-51-5	HMDB0003352	C00400	81	27.9	1690	1633+-19	1668	2	1.71	1	2.61 × 10^− 02^	84.3
MET_416	Octane, 1,1’-oxybis-	629-82-3	HMDB0061856	ND	43	29.4	1757	1761+-3	1754	1	4.57	-1	2.10 × 10^− 31^	92.9
MET_456	Ethanol, 1-(2-butoxyethoxy)-	112-34-5	HMDB0244918	ND	57	31	1829	1796+-10	1822	1	1.74	1	5.15 × 10^− 10^	92.3
MET_458	Butanenitrile, 4-(methylthio)-	59121-24-3	HMDB0302370	ND	61	31	1829	1770+-37	1835	1	1.68	1	5.97 × 10^− 10^	90.4
MET_468	2 H-pyran-2-one, tetrahydro-6-methyl-	823-22-3	HMDB0000453	ND	70	31.4	1847	1791+-20	1855	1	1.01	1	1.66 × 10^− 03^	90.6
MET_544	Phenylethyl alcohol	60-12-8	HMDB0033944	C05853	91	33.8	1962	1906+-15	1955	1	1.18	1	3.00 × 10^− 04^	96.3
MET_563	Creosol	93-51-6	HMDB0032136	ND	138	34.4	1993	1956+-12	1998	1	1.03	1	2.33 × 10^− 03^	96.5
MET_582	Propane, 1-isothiocyanato-3-(methylthio)-	505-79-3	HMDB0034370	ND	101	35.1	2028	1975+-5	2037	1	1.2	-1	5.58 × 10^− 03^	93.4
MET_588	Isopropyl tetradecanoate	110-27-0	HMDB0040392	ND	43	35.6	2050	2027+-7	2045	1	2.16	-1	5.68 × 10^− 06^	97.6
MET_600	1-phenoxypropan-2-ol	770-35-4	HMDB0341215	ND	94	36.2	2083	ND	2084	1	1.68	1	1.08 × 10^− 03^	91.6
MET_614	p-cresol	106-44-5	HMDB0001858	C01468	107	36.9	2117	2080+-12	2116	1	1.34	1	1.22 × 10^− 02^	98.4
MET_635	Nonanoic acid	112-05-0	HMDB0000847	C01601	60	38.3	2189	2171+-17	2184	1	1.71	1	1.68 × 10^− 05^	96.7
MET_643	2-piperidinone	675-20-7	HMDB0011749	ND	99	38.3	2192	2060	2214	2	1.35	1	6.87 × 10^− 03^	93.3
MET_657	4-isopropyl-2-methylphenol	1740-97-2	HMDB0038984	ND	135	38.7	2213	2221	ND	2	1.75	-1	4.56 × 10^− 03^	97.2

These VOCs represent the most robust discriminative candidates between kidney stone formers and controls. Several of these metabolites showed clear group differences, with octane, 1,1′-oxybis- (MET_416) and 6-methyl-5-hepten-2-one (MET_152) being more abundant in controls (Supplementary Fig. S4), whereas 1-butoxy-2-propanol (MET_146) and the short- and medium-chain acids acetic (MET_212), propanoic (MET_266), and nonanoic acid (MET_635) were elevated in stone formers (Supplementary Fig. S5).

These results support the hypothesis that urinary volatile profiles differ between individuals with and without kidney stones. In particular, the identification of 32 robustly discriminating VOCs suggests the existence of metabolic signatures associated with stone formation, providing a biological basis for functional interpretation and further validation.

### Metabolic signatures of fermented food consumption

To explore potential dietary drivers of the observed metabolomic signatures, associations between urinary VOCs and fermented food intake were examined. Among all detected urinary features, twenty-eight urinary metabolites were significantly associated with fermented food intake (FDR < 0.05). Analyses of dietary associations were performed across all fermented-food subgroups in the dataset and were not restricted to metabolites associated with kidney stone status. Most metabolites showed positive associations with total fermented food consumption. Cross-comparison with stone-related metabolites identified three overlapping compounds: 3-nonen-2-one (MET_252) and two unidentified features (MET_73 and MET_680) (Table 4).

**Table 4:** Association of selected urinary metabolites with fermented food intake and kidney stone status. Twenty-eight Metabolites significantly associated with fermented food intake (FDR < 0.05 in linear regression) were subsequently tested for association with kidney stone status using logistic regression. Three metabolites fulfilled both criteria, association with fermented food consumption and discrimination between stone formers and controls. Among these, only MET_252 (3-nonen-2-one) could be chemically identified according to MSI guidelines. MET_252 showed an inverse association with kidney stone status, whereas MET_680 was positive and MET_73 inversely associated

Metabolite	*p*-value_FDR, case−control_	-log_10_(*p*)	significant	Consommation	OR	OR_low	OR_high	*p*-value_FDR, FFI_
**MET_252** **3-Nonen-2-one**	0.0023	4.167	TRUE	Fermented food intake	**0.48**	0.37	0.61	**3.1 × 10** ^**− 07**^
**MET_680** **Unknown**	0.0058	3.747	TRUE	Fermented food intake	**1.32**	1.08	1.61	**0.0088**
**MET_73** **Unknown**	0.0417	2.766	TRUE	Fermented food intake	**0.75**	0.61	0.92	**0.0089**

OR: Odds ratio. *P-value*_*FDR, case−control*_: FDR-adjusted p-value for the association between the metabolite and kidney stone status (case vs. control). *P-value*_*FDR, FFI*_: FDR-adjusted p-value for the association between the metabolite and Fermented Food Intake (dietary exposure)

**Fig. 3:**
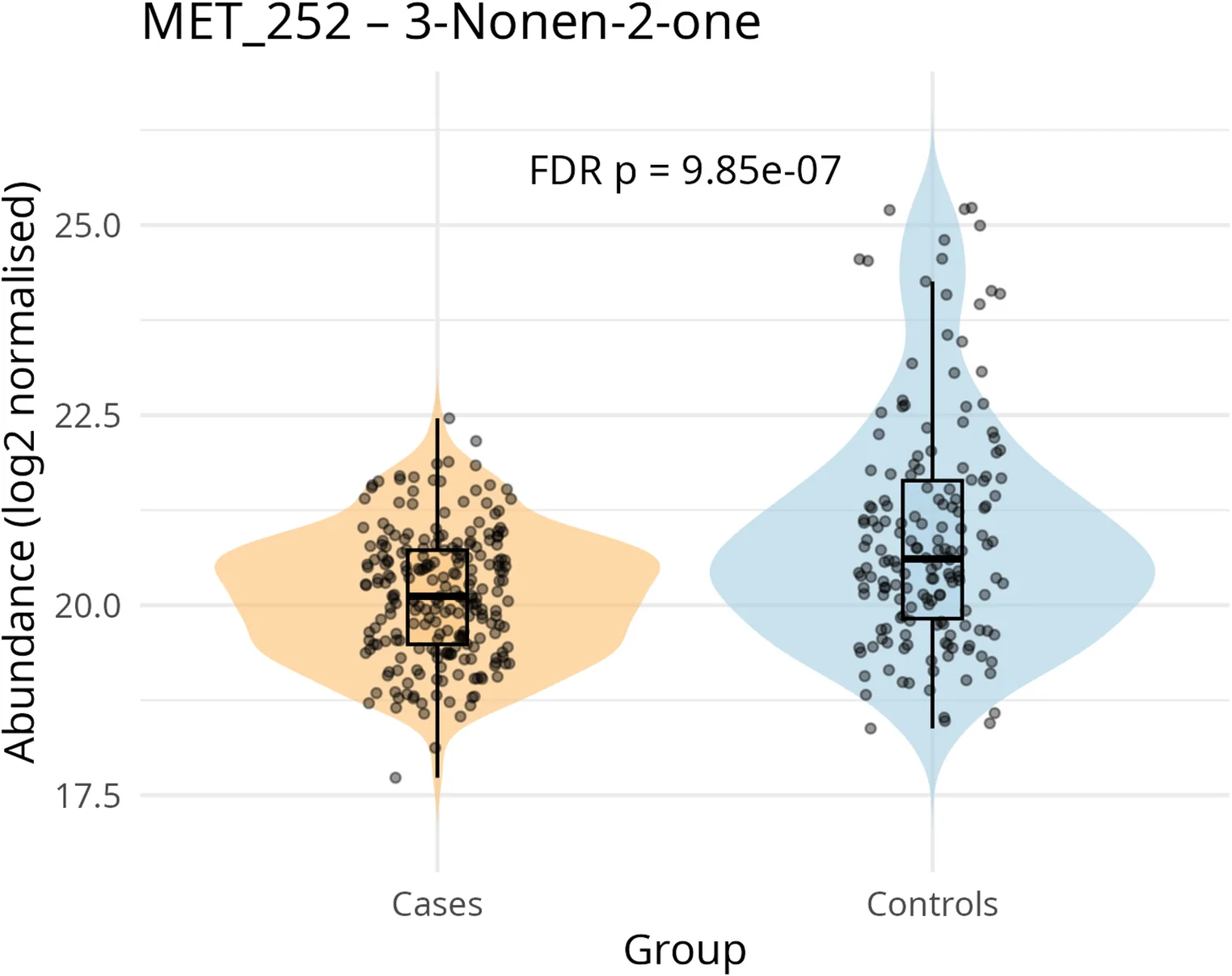
Violin plots of log₂-normalized urinary 3-nonen-2-one abundance in kidney stone cases (orange) and controls (blue). The figure displays the distribution of metabolite levels across groups, with median values and density shapes illustrating inter-individual variability. Statistical significance of group differences is reported in the text

Notably, 3-nonen-2-one (Fig. 3) showed a strong inverse association with kidney stone status (OR = 0.48, FDR, FFI = 3.1 × 10^− 7^) and identification was confirmed at MSI level 1. To clarify, dietary association analyses were restricted to the metabolites that significantly differentiated cases from controls. Within this framework, subgroup analyses showed that coffee intake (subgroup “1_3_Coffee”, **Supplementary Table ****S1**) was the predominant predictor of urinary 3-nonen-2-one (FDR = 0.0023).

**Fig. 4:**
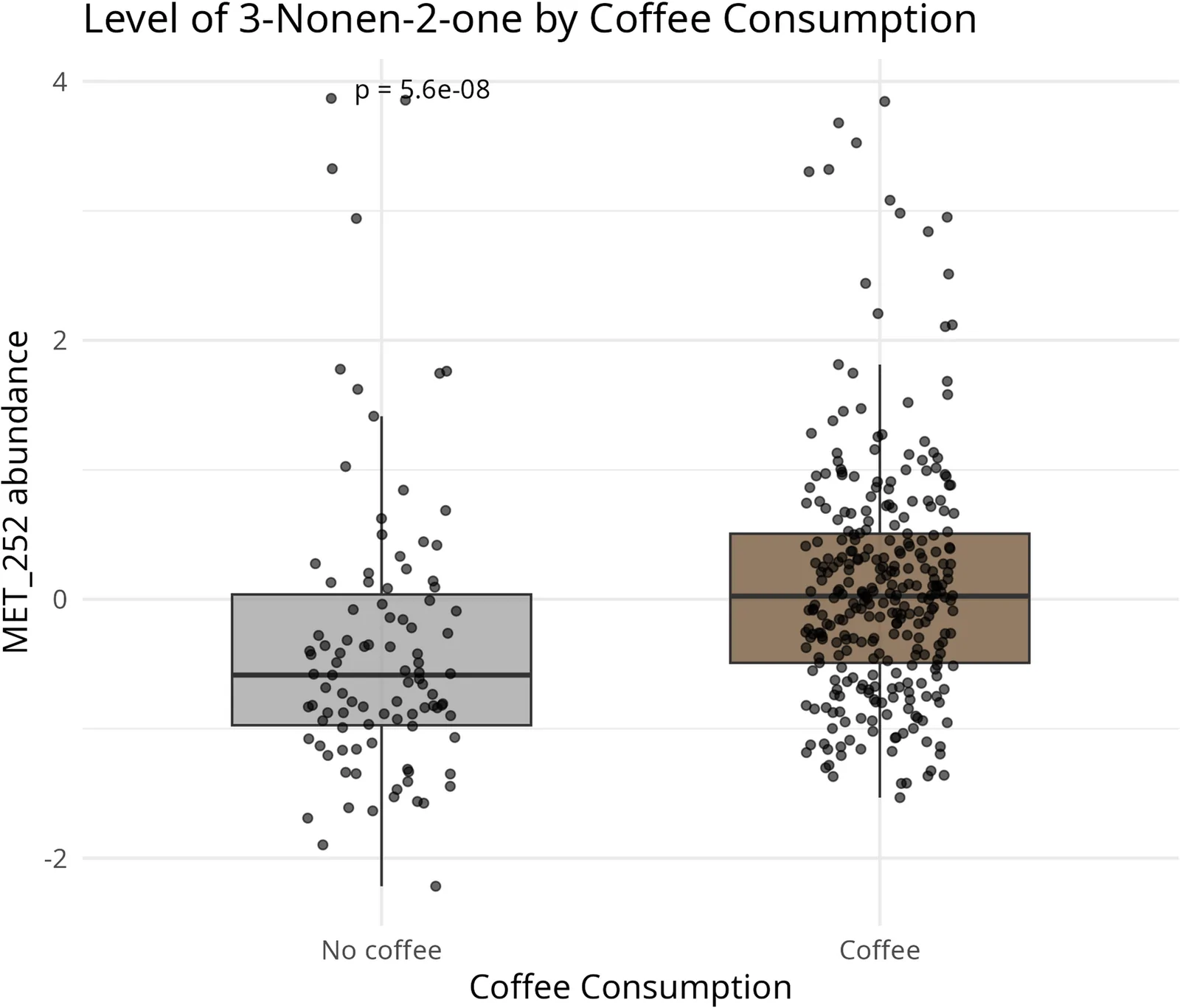
Boxplot of log-transformed urinary 3-nonen-2-one abundance according to coffee consumption status. The figure displays the distribution of metabolite levels among coffee consumers and non-consumers, illustrating group medians, interquartile ranges, and outliers

Individuals consuming coffee displayed substantially higher urinary concentrations of 3-nonen-2-one (*p* = 5.6 × 10^− 8^, Fig. 4). Given the observed associations between coffee intake, urinary 3-nonen-2-one, and kidney stone status, an exploratory causal mediation analysis was performed. The results are summarized in Table 5.

**Table 5:** Exploratory mediation analysis of coffee intake, 3-nonen-2-one, and kidney stone risk. The mediation model showed a statistically significant indirect effect (ACME) through urinary 3-nonen-2-one, suggesting that part of the association between coffee intake and kidney stone status may operate via this metabolite. The direct effect (ADE) was not significant, and approximately 59% of the total estimated association was attributed to the indirect pathway

Parameter	Estimate	95% CI	*p*-value	Interpretation
**ACME (Indirect effect)**	**-0.0654**	[-0.116 ; -0.020]	**< 2e** ^**− 16**^	**Significant indirect association consistent with mediation through urinary 3-nonen-2-one**
**ADE** **(Direct effect)**	-0.0457	[-0.158 ; 0.070]	0.492	Not significant
**Total effect**	-0.1111	[-0.235 ; 0.000]	0.062	The overall effect is borderline
**Proportion mediated**	58.8%	[-107.2% ; 355%]	0.062	A substantial proportion of the observed association was statistically attributed to the indirect pathway, although the confidence interval was wide and included uncertainty.

ACME: Average causal mediation effect. ADE: Average direct effect. CI: Confidence interval

### KEGG-based metabolic pathway analysis

Using KEGG compound identifiers [28], three metabolic pathways were prominently associated with the detected metabolites. The highest-ranked pathways were Propanoate metabolism (hsa00640), Protein digestion and absorption (hsa04974), and Alcohol metabolism (hsa05034). These pathways were highlighted by the presence of multiple interconnected metabolites within the KEGG metabolic network (Supplementary Table S3).

Propanoate metabolism was driven by short-chain fatty acid–related metabolites, including propanoate, propanal, acetate, and acetone, which mapped to reactions involving CoA-ligases and oxidoreductases.

Protein digestion and absorption emerged due to aromatic amino acid–derived metabolites such as p-cresol and phenylethyl alcohol, which are downstream products of tyrosine and phenylalanine degradation. Although KEGG links these metabolites to digestive peptidases, no enzymes were measured in this study.

Alcohol metabolism was supported by volatile alcohol (e.g., ethanol, propanol, phenylethyl alcohol) connected to classical alcohol and aldehyde dehydrogenase pathways. Their simultaneous detection accounted for the enrichment of this metabolic network.

## Discussion

We identified a distinct urinary volatile signature differentiating kidney stone formers from healthy controls. Several metabolites showed the strongest and most consistent discrimination, including 1-butoxy-2-propanol, acetic acid, propanoic acid, and nonanoic acid, which were elevated in cases, while octane, 1,1’-oxybis- and 6-methyl-5-hepten-2-one were more abundant in controls. Notably, 3-nonen-2-one also emerged as a key discriminant metabolite, showing a strong inverse association with kidney stone status and displaying clear association with coffee consumption. Together, these VOCs constitute a characteristic metabolic signature distinguishing stone formers from controls. Collectively, our findings indicate that nephrolithiasis is associated with a complex metabolic signature, potentially shaped by oxidative stress, endogenous acid production, and environmental exposures influencing urinary chemistry. Several volatile metabolites differentiated stone formers from controls (77 metabolites in total), and their biological profiles align with metabolic and physiological changes previously implicated in stone disease, as identified using metabolomics approaches in human and animal studies of urolithiasis [30]. 

### Confounder adjustment

Study center and sex emerged as key sources of variability in urinary VOC profiles, in line with previous reports showing site-related technical effects and known sex-dependent metabolic differences [31].

Additional variables such as age, BMI, smoking status, and diabetes may also influence specific metabolites and were therefore considered in the statistical adjustment strategy.

Correcting for these covariates was essential to ensure that subsequent analyses reflected biological differences related to kidney stone status rather than technical variation or confounding factors. This approach assumes these variables act as confounders rather than effect modifiers; although interactions, for example with sex or age, could theoretically influence metabolite–disease associations, investigating such effect modification was beyond the scope of this analysis. By minimizing the impact of unwanted variability, the adjusted dataset provides a more reliable basis for interpreting disease-related metabolic signals in downstream multivariate analyses.

### Identification of discriminative metabolites

Validation of the OPLS-DA model using the confounder adjusted dataset confirmed that the case–control separation was not driven by confounding or technical artifacts. Although R²Y and Q² values were slightly lower than in the unadjusted model, this reflects a more conservative and accurate representation of the biological signal. Permutation testing of the class labels (stone formers vs. controls) within the OPLS-DA framework (*n* = 999 permutations) showed that the observed model performance was significantly better than would be expected by chance (*p* = 0.001), thereby supporting the presence of genuine metabolic differences between groups. VIP-based feature selection initially produced a redundant set of metabolites due to the automated GC–EI–MS deconvolution process. Manual review removed overlapping or non-biological signals, yielding a filtered and biologically interpretable set of metabolites. This filtered set revealed clear metabolic differences between stone formers and controls, and the integrated OPLS-DA and FDR-adjusted Wilcoxon analyses identified a subset of potential biomarkers reliably associated with disease status.

### Biological interpretation

More broadly, the identified VOCs were grouped into functional categories by examining their known biochemical roles and previously reported associations with inflammation, oxidative stress, and metabolic processes relevant to kidney stone formation. Several volatile metabolites distinguished stone formers from controls, reflecting endogenous, gut microbial activity (e.g., short-chain volatile acids such as acetic and propanoic acid, derived from microbial fermentation pathways), and exogenous exposure–related processes. Healthy participants showed higher levels of octane, 1,1′-oxybis-, whose physiological relevance remains unclear. This compound has previously been detected in healthy individuals and during pregnancy, suggesting endogenous production or excretion [32, 33]. It has also been reported in fresh non-alcoholic beer, where its disappearance after processing indicates sensitivity to oxidation [34]. Although its biological role remains uncertain, its consistent detection in controls suggests that it may represent a candidate biomarker deserving further investigation.

6-Methyl-5-hepten-2-one (sulcatone), an oxidative derivative of squalene, was more abundant in controls. Because squalene oxidation is closely linked to oxidative stress [35], lower urinary sulcatone concentrations in stone formers may reflect alterations in redox homeostasis, although this interpretation remains speculative. Acetic, propanoic and nonanoic acids were elevated in kidney stone formers. Acetic and propanoic acids may reflect altered microbial fermentation and inflammatory processes [36], whereas nonanoic acid has been associated with oxidative stress and impaired fatty-acid metabolism [37–41]. Together, these metabolites support the hypothesis of altered microbial and lipid metabolism in nephrolithiasis, although causal relationships cannot be established.

1-Butoxy-2-propanol was markedly elevated in cases. This glycol ether is widely used in industrial and household products and is metabolized through alcohol- and aldehyde-dehydrogenase pathways [42]. Although its role in kidney stone formation remains uncertain, it may primarily represent a marker of environmental exposure. More broadly, several discriminatory VOCs identified in this study, including glycol ethers and related compounds, may originate from environmental or occupational exposures. As urinary volatilomics reflects both endogenous metabolism and external exposures, these compounds should be interpreted as candidate biomarkers associated with kidney stone status rather than disease-specific metabolites. Further studies integrating detailed exposure assessment are needed to clarify their biological origin and relevance to kidney stone disease.

The KEGG pathway enrichment analysis suggests that several detected metabolites may map to pathways related to propanoate metabolism, alcohol metabolism, and protein digestion. The metabolites associated with propanoate metabolism could reflect processes involving short-chain fatty acid turnover, although this interpretation should remain cautious, as pathway enrichment is indirect and short-chain fatty acids were not directly quantified in this study. The presence of volatile alcohols and their oxidized derivatives appears compatible with activity within classical alcohol and aldehyde dehydrogenase pathways, without allowing conclusions regarding their origin. In addition, the aromatic metabolites observed (e.g., p-cresol, phenylethyl alcohol) may reflect downstream products of tyrosine- and phenylalanine-related degradation pathways, although their specific metabolic origin cannot be determined from the present data.

Overall, these pathway signals should be interpreted as potential indicators of metabolic context rather than definitive evidence of specific biochemical mechanisms.

To facilitate interpretation of the findings, the main characteristics and proposed biological relevance of the principal VOCs discussed above are summarized in Table 6.

**Table 6:** Summary of the main biological interpretation of the most relevant VOCs associated with kidney stone disease. Summary of the principal VOCs discussed in the biological interpretation section. Relative abundance refers to kidney stone formers compared with healthy controls

VOC	Relative abundance	Proposed biological source	Potential biological interpretation	Strength of current evidence
3-Nonen-2-one	↓ Kidney stone formers	Coffee-derived metabolite and/or lipid metabolism	Candidate metabolite potentially mediating the observed association between coffee consumption and lower kidney stone risk	Moderate
Sulcatone (6-methyl-5-hepten-2-one)	↓ Kidney stone formers	Lipid peroxidation and squalene oxidation	May reflect altered oxidative stress and antioxidant status	Moderate
Acetic acid	↑ Kidney stone formers	Gut microbial fermentation	May indicate altered microbial metabolism and inflammatory processes	Moderate
Propanoic acid	↑ Kidney stone formers	Gut microbial fermentation	May reflect alterations in microbial short-chain fatty acid metabolism and host-microbiota interactions	Moderate
Nonanoic acid	↑ Kidney stone formers	Fatty acid metabolism	May be associated with altered lipid metabolism and oxidative stress	Low–Moderate
Octane, 1,1’-oxybis-	↓ Kidney stone formers	Unknown	Biological significance remains unclear; potential candidate biomarkers requiring further investigation	Low

### Metabolic effects of fermented food consumption

Habitual consumption of fermented foods was associated with distinct urinary metabolic profiles, indicating that regular consumers of these foods exhibit specific metabolite patterns. This finding is consistent with recent work using National Health and Nutrition Examination Survey (NHANES) dietary data, in which foods were classified according to their fermentation status and microbial content. These analyses suggest that both viable microbes and non-viable fermentation-derived components (such as bioactive metabolites or fermentation end products) may influence host metabolism [43]. In the context of kidney stones, our interpretation specifically refers to the subset of fermentation-related metabolites that also showed associations with kidney-stone status, indicating that both fermentation-derived compounds and downstream dietary metabolites may contribute to metabolic variation relevant to stone pathophysiology. Out of the metabolites associated with fermented food intake (*n* = 28), three (3-nonen-2-one, MET_73, MET_680) were also associated with kidney stone status. Only 3-nonen-2-one could be identified according to MSI criteria. 3-nonen-2-one and MET_73 were inversely associated with lithiasis, whereas MET_680 showed a positive association. This overlap indicates that only a few fermentation-related metabolites were also associated with kidney stone status in our dataset. 3-Nonen-2-one emerged as a potential and confidently identified metabolite (MSI Level 1), showing consistent associations with both fermented food intake and coffee consumption (FDR = 0.0023). Coffee, a known source of volatile organic compounds formed during roasting, was the only food group significantly linked to 3-nonen-2-one levels [44]. Some non-consumers also exhibited elevated levels of 3-nonen-2-one, which may reflect unreported or indirect coffee intake (e.g., pastries or sweets containing coffee) or the presence of other dietary sources of 3-nonen-2-one did not assess in this study. This observation suggests that in addition to dietary exposure (via coffee), there may be other sources of this ketone, for example endogenous metabolic production or microbial transformation. To our knowledge, there is currently no published data detailing a human metabolic or microbiome pathway specifically generating 3-nonen-2-one. Therefore, these alternate sources remain speculative and warrant further investigation.

The exploratory mediation analysis suggests that urinary 3-nonen-2-one may partly mediate the observed association between coffee intake and lower kidney stone risk. However, given the observational and cross-sectional nature of the present study, these findings should be considered hypothesis-generating and require confirmation in independent longitudinal and mechanistic studies. The direct effect of coffee alone was not statistically significant, implying that this metabolite partly mediates the protective relationship.

These results align with large-scale epidemiological studies reporting a 19%–40% reduction in kidney stone risk associated with coffee or caffeine intake [45, 46]. Consistently, Legay et al. [7] also reported lower coffee consumption among stone formers in the SKSC cohort, supporting the notion that reduced intake may be characteristic of individuals at higher risk. Mechanistically, 3-nonen-2-one can undergo reduction by prostaglandin reductase 1 (PGR1), an NADPH-dependent oxidoreductase known for its high catalytic efficiency toward α, β-unsaturated ketones [47]. This enzymatic pathway likely contributes to the detoxification or metabolic turnover of 3-nonen-2-one in human tissues. However, the observation of elevated urinary levels of 3-nonen-2-one in apparently healthy individuals suggests that additional mechanisms may be involved. One possibility is that PGR1 activity becomes saturated under certain metabolic conditions, limiting further reduction. Alternatively, regulatory mechanisms could maintain higher circulating or urinary levels of 3-nonen-2-one to fulfill yet-unknown physiological or signaling roles. These interpretations should be considered hypothetical, as current evidence does not allow their experimental verification. Further experimental studies are needed to determine whether these elevations reflect enzyme kinetics, substrate availability, or adaptive metabolic control.

Collectively, these findings identify 3-nonen-2-one as a candidate metabolite that may partly mediate the observed association between coffee consumption and kidney stone risk, although this hypothesis requires validation in independent and longitudinal studies.

### Strengths and limitations

This study has several strengths, including the use of a well-characterized multicenter cohort, standardized urine collection and dietary assessment, and comprehensive untargeted urinary volatilomic profiling. Nevertheless, several limitations should be considered. First, from an epidemiological perspective, the SKSC represents a convenience sample, since recruitment was hospital-based and disease-driven and the control group was not representative of the general population. The results should therefore be interpreted with caution regarding external validity. Although the Swiss Kidney Stone Cohort provides a well-characterized population with standardized clinical, dietary, and metabolomic data collection, dietary habits, genetic background, and environmental exposures may differ across populations. Consequently, the generalizability of these findings to other geographic or ethnic populations remains to be established [26]. Second, although major confounders were controlled for, residual confounding cannot be excluded. Cases and controls were not matched for age and sex. Finally, the cross-sectional design limits causal inference.

In addition, several limitations specific to the present analysis should be acknowledged. Dietary intake was assessed using standardized interviewer-administered 24-h dietary recalls, which may not fully reflect participants’ habitual dietary patterns. Information on medication use, vitamin supplementation, and other dietary supplements was not available for the present analysis. These unmeasured factors may have influenced urinary metabolomic profiles and therefore residual confounding cannot be excluded. Furthermore, stone composition was not systematically determined in our cohort. Although calcium oxalate stones (both monohydrate and dihydrate) are the most prevalent stone types in adults and therefore likely represent most cases, the study population may also have included uric acid, calcium phosphate, or other stone subtypes with distinct underlying biological mechanisms. This biological heterogeneity may have influenced the urinary VOC profiles independently of stone status and therefore represents an important potential confounder when interpreting the identified metabolomic signatures. Moreover, the present findings were obtained in a single Swiss cohort. Validation in independent cohorts from diverse geographic regions, with different dietary habits and genetic backgrounds, will therefore be necessary before these VOCs can be considered robust biomarkers of kidney stone disease. The present study was designed as a biomarker discovery study rather than a diagnostic validation study. Although several urinary VOCs discriminated kidney stone formers from controls, the present data do not allow conclusions regarding their clinical diagnostic performance. Future studies should evaluate the sensitivity, specificity, predictive values, optimal decision thresholds, and external validation of these candidate biomarkers in independent prospective cohorts before considering their implementation as clinical diagnostic tools.

In addition, the DHS-V-ITEX-GC-MS platform used in this study is intended for biomarker discovery and is not routinely available in clinical laboratories. Following validation, the identified VOC biomarkers could potentially serve as targets for the development of simplified analytical assays or point-of-care (POC) sensing technologies suitable for routine clinical use.

## Conclusion

In this observational study, urinary volatilomic profiling revealed differences in volatile organic compound patterns between kidney stone formers and healthy individuals. Several metabolites were associated with kidney stone status, suggesting that metabolic pathways related to oxidative processes, organic acid metabolism, microbial activity, and environmental exposure may contribute to the observed variability. Among the detected compounds, 3-nonen-2-one showed consistent associations with coffee consumption, and kidney stone status, indicating a potential link between diet-related metabolites and stone risk. Although causal relationships cannot be inferred, these findings provide descriptive evidence that urinary volatile metabolites may reflect metabolic and dietary factors relevant to nephrolithiasis. Although the mediation analysis is consistent with a potential intermediary role of 3-nonen-2-one, this finding should be considered exploratory and hypothesis-generating and requires confirmation in independent longitudinal and mechanistic studies. Future studies should validate these candidate biomarkers in independent cohorts with different dietary habits, genetic backgrounds, and environmental exposures. Longitudinal and mechanistic studies will also be required to clarify their biological significance and potential clinical utility.

Taken together, these findings support the potential of urinary volatilomics as a complementary approach for biomarker discovery in kidney stone disease.

## Supplementary Information

Below is the link to the electronic supplementary material.

Supplementary Material 1

## Data Availability

All data are accessible upon request to authors.
