# Supplementary material for: Urinary volatilomics for biomarker discovery in kidney stone patients and healthy controls
Source: Urolithiasis. 2026 Aug 25;54(1):137. doi: 10.1007/s00240-026-02045-7 (PMC13506514; doi:10.1007/s00240-026-02045-7)
Supplement: Supplementary file 1 [file 240_2026_2045_MOESM1_ESM.docx]

**Supplementary appendices**

**Urinary volatilomics for biomarker discovery in kidney stone patients and healthy controls**

*Pascal Fuchsmann*

**table of contents**

[Supplementary methods](#_Toc214551090)

[Chemicals and Handling…………………………………………………………………	2](#_Toc214551091)

[Sample Preparation for GC-MS Analysis………………………………………………	2](#_Toc214551092)

[Normalization, imputation, and scaling…………………………………………………	3](#_Toc214551093)

[Statistical analysis………………………………………………………………………..	4](#_Toc214551094)

[Metabolite Chemical Identification According to MSI Guidelines……………………	4](#_Toc214551095)

[Fermented food intake mediation analysis…………………………………………….	4](#_Toc214551096)

**Supplemental Figures**

[**Supplementary Fig. S1:** Workflow for GC-MS data processing and biomarker discovery.	6](#_Toc214451461)

[**Supplementary Fig. S2:** PLS-DA score plots illustrating the influence of potential confounders on urinary VOC profiles.	7](#_Toc214451462)

[**Supplementary Fig. S3:** OPLS-DA score plots illustrating the effect of data correction on group separation between kidney stone cases (blue) and controls (red).	8](#_Toc214451463)

[**Supplementary Fig. S4:** Violin plots of urinary octane, 1,1′-oxybis- (left) and 6-methyl-5-hepten-2-one (MET_152, right) in kidney stone cases (orange) and controls (blue).	9](#_Toc214451464)

[**Supplementary Fig. S5:** Violin plots of log₂-normalized urinary abundances of acetic (A), propanoic (B), and nonanoic acid (C), and 1-butoxy-2-propanol (D) in kidney stone cases (orange) and controls (blue).	11](#_Toc214451465)

**Supplemental Tables**

[**Supplementary Table S1:** Food intake categories used in dietary data analysis.	12](#_Toc214451466)

[**Supplementary Table S2:** Volatile organic compounds (VOCs) identified as potential biomarkers by GC–MS.	13](#_Toc214451467)

**Supplementary Table S3:** Complete KEGG/FELLA Output for Pathway Enrichment Analysis…………………………………………………………………………...……...17

# **Supplementary methods: GC–MS Analysis and Volatilomic Data Processing**

## **Chemicals and Handling**

Urine samples (n = 400) were obtained from the Swiss Kidney Stone Cohort biobank. For the present study, only native urine aliquots without thymol preservative were used. Samples had been stored in polypropylene cryogenic tubes at −80 °C until analysis. Each aliquot underwent a single freeze–thaw cycle. Samples were removed individually from the −80 °C freezer, thawed on ice until completely liquid, vortex-mixed, and immediately transferred into headspace vials to minimize volatile losses. After thawing, 250 µL were removed for analysis and specific gravity was measured using an RE40 refractometer (Mettler Toledo, Bussigny, Switzerland).

## **Sample Preparation for GC-MS Analysis**

Automated preparation was performed using a Hamilton Microlab STAR robot (Hamilton Company, Bonaduz, Switzerland). For each sample, 100 µL urine was acidified with 10 µL of 1% H₃PO₄ and transferred to 20 mL headspace vials. Quality-control (QC) samples were prepared by pooling 100 µL from all specimens. Each batch began with blanks and five QCs, with additional QC injections every 10 samples. Case and control samples were randomized to avoid run-order effects. Urine aliquots were transferred immediately after opening the cryovials into 20 mL headspace vials, which were sealed immediately after pipetting. Prepared headspace vials were refrozen (-40°C) until analysis and subsequently maintained at 4 °C in the autosampler during the analytical sequence, thereby minimizing headspace losses and volatilization before DHS-V-ITEX extraction.

**Analytical platform and DHS-V-ITEX extraction**

GC–MS analyses were performed on an Agilent 7890B GC coupled to a 5977B HES MS (Agilent Technologies, Basel Switzerland). Vacuum in-tube extraction (V-ITEX) was conducted as described by Fuchsmann et al. (16). Samples were incubated at 80 °C for 2 min and extracted for 5 min under 5 mbar vacuum. The ITEX trap (Tenax TA/Carbosieve SIII, 60/80 mesh) was held at 35 °C; the syringe at 80 °C. Desorption occurred at 300 °C in a CIS4 injector (Gerstel AG, Sursee, Switzerland) operated in vent mode.

Compounds were separated on a 60 m × 0.25 mm, 0.5 µm Optima FFAP-Plus column (Macherey-Nagel, Oensingen, Switzerland). Oven: 40 °C (5 min), ramp 5 °C/min to 240 °C, hold 9 min (total: 52 min). MS acquisition was SCAN (*m/z* 29–350).

**Data processing workflow**

Raw data were processed with MassHunter Profinder 10.0 (Agilent). Agile2 (Agilent algorithm) deconvolution was used for peak picking. Features were extracted within 3.5–23 min RT, with minimum intensity thresholds of 400 counts (absolute) and 0.005 (relative). Only features present in ≥10% of samples in at least one group were retained.

Retention-time alignment and targeted feature extraction were performed to generate consolidated compound tables. A modified “80% rule” was applied whereby features present in ≥80% of samples within each group (cases or controls) were kept (1).

## **Normalization, imputation, and scaling**

Data were normalized sequentially using probabilistic quotient normalization (PQN) and LOESS correction based on QC injections (2). Locally estimated scatterplot smoothing (LOESS) normalization, a local regression smoothing method, was then applied using QC samples injected throughout the analytical sequence to correct for batch effects and instrument drift over time (2).

Features present in <80% of samples within either group were excluded prior to imputation. Because this filtering step removed low-presence features, no additional missingness threshold was applied before random forest imputation (3).

Data were log_2_-transformed and autoscaled prior to statistical modelling (4).

## **Statistical analysis**

Covariate adjustment (age, BMI, diabetes, sex, smoking, center) was performed using linear regression (4). Univariate comparisons between groups used Wilcoxon rank-sum tests with FDR correction (Benjamini–Hochberg).

Multivariate analyses (PLS-DA, OPLS-DA) identified discriminant metabolites; variables were ranked using VIP scores. Cross-validation and permutation tests assessed model robustness. Feature stability was evaluated by bootstrap resampling. A schematic workflow is provided in **Supplemental** **Figure 1**.

## **Metabolite Chemical Identification According to MSI Guidelines**

Identification combined spectral matching (NIST23 library) and retention indices (RI) determined using a C_6_-C_30_ alkane series. When available, reference standards processed under identical conditions supported identification. Confidence levels followed Metabolomics Standards Initiative categories (5-7):

**Level 1**: spectral match ≥90% and RI within ±10–15 units + standard confirmation

**Level 2**: match >80%, RI within ±15, no standard

**Level 3**: putative class-level identification

**Level 4**: unknowns

## **Fermented food intake mediation analysis**

A causal mediation analysis was performed to determine whether the association between the dietary exposure of interest, specifically, coffee intake within the fermented-food subgroup, and kidney stone status was mediated through the selected urinary metabolite. The analysis was implemented using the mediation package in R (8) following the counterfactual framework proposed by Imai et al. (9).

Two regression models were fitted: (i) a linear regression assessing the effect of the dietary exposure on the mediator, and (ii) a logistic regression assessing the joint effects of the exposure and the mediator on the binary outcome (kidney stone status). The average causal mediation effect (ACME), average direct effect (ADE), and total effect were estimated using nonparametric bootstrapping with 5,000 simulations to produce bias-corrected confidence intervals.

All models were adjusted for the covariates identified as potential confounders in the main analysis. The proportion mediated was calculated as ACME divided by the total effect. Statistical significance was defined as p < 0.05 (two-sided).

# **Supplementary Figures**

**Supplementary Fig. S1** Workflow for GC-MS data processing and biomarker discovery.

The figure illustrates the stepwise pipeline for processing urinary VOC data. The steps include GC-MS data preprocessing, normalization (PQN and LOESS), imputation of missing values using random forest, log2 transformation and autoscaling, and confounder and sample imbalance correction, followed by univariate and multivariate statistical analyses, and biological validation.

|   A: Sex effect |   B: Smoking status effect |
| --- | --- |
|   C: Study center effect |   D: Age effect |
|   E: BMI effect |   F: Diabetes status |

**Supplementary Fig. S2** PLS-DA score plots illustrating the influence of potential confounders on urinary VOC profiles.

Each panel (A–F) displays samples colored according to confounder category, with ellipses representing 95% confidence regions. Panels correspond to: (A) sex, (B) smoking status, (C) study center, (D) age, (E) BMI, and (F) diabetes status. The plots visualize potential group structure and variance attributable to each variable.

|  |  |
| --- | --- |

**Supplementary Fig. S3** OPLS-DA score plots illustrating the effect of data correction on group separation between kidney stone cases (blue) and controls (red).

The left panel shows the model based on uncorrected data (one predictive and three orthogonal components; pre = 1, ort = 3). The right panel presents the corrected model (pre = 1, ort = 2) after confounder adjustment. Model performance parameters (R²X, R²Y, Q²Y, RMSEE) are reported within each panel. The figure demonstrates how preprocessing and confounder adjustment influence model structure and class discrimination.

|  |  |
| --- | --- |

**Supplementary Fig. S4** Violin plots of urinary octane, 1,1′-oxybis- (left) and 6-methyl-5-hepten-2-one (MET_152, right) in kidney stone cases (orange) and controls (blue).

The figure displays log₂-normalized metabolite abundances for each group. Dots represent individual samples, and box elements indicate medians and interquartile ranges. Statistical comparisons were performed using FDR-adjusted Wilcoxon rank-sum tests (results reported in the text).

|   A |   B |   C |
| --- | --- | --- |
|   D |  |  |

**Supplementary Fig. S5** Violin plots of log₂-normalized urinary abundances of acetic (A), propanoic (B), and nonanoic acid (C), and 1-butoxy-2-propanol (D) in kidney stone cases (orange) and controls (blue).

Each dot represents an individual sample; central boxes indicate medians and interquartile ranges. The y-axis represents unitless log₂-normalized abundance. Statistical significance was evaluated using FDR-adjusted Wilcoxon rank-sum tests (p-values reported in the text).

# **Supplementary tables**

**Supplementary Table S1** Food intake categories used in dietary data analysis.

| 1_1_Water | 4_19_Other protein-based products |
| --- | --- |
| 1_2_Tea | 5_20_Vegetable oil |
| 1_3_Coffee | 5_21_Butter |
| 2_4_Vegetables | 5_22_Cream, Fatty sauces, Other fats |
| 2_5_Fruit | 5_23_Nuts, Seeds, Olives |
| 2_6_100% juices | 6_24_Added sweeteners |
| 3_7_Tuber products | 6_25_Cakes, Desserts, Ice cream |
| 3_8_Bread products | 6_26_Chocolate products |
| 3_9_Pasta, Rice | 6_27_Other sweet products |
| 3_10_Other cereal products | 6_28_Salty snacks |
| 4_11_Milk | 6_29_Soft drinks |
| 4_12_Yoghurt, Fresh cheese | 6_30_Beer & Cider |
| 4_13_Soft cheese | 6_31_Wine |
| 4_14_Hard cheese | 6_32_Other alcohols |
| 4_15_Red meat | 6_33_Fortified wines, Liqueurs, Spirits |
| 4_16_Other unprocessed meat | 6_34_Condiments and seasonings, including sauces, herbs, spices |
| 4_17_Processed meat | 6_35_Artificial sweeteners |
| 4_18_Fish, Seafood |  |

Dietary information was collected using 24-hour dietary recalls. Each entry lists a numeric code and the corresponding food group name. The first digit identifies the main food category, and the second digit denotes its sub-category.

**Supplementary Table S2** Volatile organic compounds (VOCs) identified as potential biomarkers by GC–MS.

| **Feature_ID** | **Compound name** | **CAS N°** | **HMDB N°** | ***m/z*** | **RT** | **Retention index calculated** | **Retention index literature** | **Retention index standards** | **Level of identification 1-4** | **VIP pred** | **delta** | **Direction** | **wilcoxon_pvalue**  **FDR** | **Match** |
| --- | --- | --- | --- | --- | --- | --- | --- | --- | --- | --- | --- | --- | --- | --- |
| MET_05 | Propanal | 123-38-6 | HMDB0003366 | 58 | 4.02 | 725 | 798+-14 | 780 | 2 | 1.7 | -0.56 | -1 | 5.1E-06 | 83.2 |
| MET_06 | Ethanol | 64-17-5 | HMDB0000108 | 46 | 4.05 | 727 | 932+-8 | 941 | 2 | 1.7 | -0.41 | -1 | 1.8E-04 | 95.3 |
| MET_11 | Acetone | 64-17-5 | HMDB0001659 | 43 | 4.18 | 734 | 832 | 805 | 2 | 2.1 | -0.61 | -1 | 3.9E-07 | 95.3 |
| MET_30 | 3-buten-2-one | 78-94-4 | HMDB0061873 | 70 | 7.12 | 963 | 946+- 4 | 952 | 1 | 1.6 | 0.41 | 1 | 1.2E-01 | 96.6 |
| MET_38 | 2-pentanone | 107-87-9 | HMDB0034235 | 85 | 7.99 | 994 | 981+-11 | 988 | 1 | 1.2 | -0.31 | -1 | 1.2E-02 | 91.9 |
| MET_53 | Furan, 2,3,5-trimethyl- | 10504-04-8 | HMDB0029721 | 110 | 10.55 | 1076 | 1065 | ND | 2 | 1.3 | 0.31 | 1 | 3.7E-03 | 85.8 |
| MET_54 | 2,3-pentanedione | 600-14-6 | HMDB0031598 | 43 | 10.74 | 1076 | 1067 | ND | 1 | 1.4 | 0.34 | 1 | 1.4E-03 | 95.6 |
| MET_66 | (R)-(-)-2-pentanol | 31087-44-2 | HMDB0031599 | 45 | 12.58 | 1133 | 1130 | 1128 | 1 | 1.6 | -0.32 | -1 | 3.2E-04 | 88.7 |
| MET_69 | Unknown 1 | NA | NA | 57 | 12.82 | 1141 | NA | ND | 4 | 1.9 | -0.40 | -1 | 1.8E-03 | 0.0 |
| MET_70 | 2-propanol, 1-methoxy- | 107-98-2 | HMDB0032494 | 45 | 12.85 | 1142 | 1045 | 1139 | 1 | 1.7 | -0.31 | -1 | 2.2E-01 | 87.8 |
| MET_73 | Unknown 2 | NA | NA | 43 | 12.95 | 1145 | NA | ND | 4 | 1.2 | -0.28 | -1 | 9.7E-02 | 0.0 |
| MET_76 | 3-methylcyclopentyl acetate ^t^ | 24070-70-0 | NA | 43 | 13.98 | 1178 | NA | ND | 3 | 1.1 | -0.27 | -1 | 1.3E-02 | 93.3 |
| MET_90 | α-Phellandrene) ^t^ | 99-83-2 | HMDB0035850 | 77 | 15.39 | 1223 | 1167+-9 | 1166 | 3 | 2.6 | -0.51 | -1 | 1.5E-06 | 87.2 |
| MET_109 | 2,6-dimethyl-1,3,5,7-octatetraene, E,E- ^t^ | 460-01-5 | NA | 133 | 16.87 | 1271 | 1460 | ND | 3 | 1.2 | 0.26 | 1 | 6.4E-02 | 89.4 |
| MET_129 | Ethanol, 2-(1-methylethoxy)-, acetate ^t^ | 108-65-6 | NA | 43 | 18.47 | 1324 | 1233+-5 | ND | 3 | 1.2 | 0.39 | 1 | 5.2E-04 | 98.5 |
| MET_146 | 2-propanol, 1-butoxy- | 5131-66-8 | NA | 45 | 19.41 | 1357 | 1353+-10 | 1352 | 1 | 2.5 | 0.74 | 1 | 1.1E-13 | 87.5 |
| MET_145 | Unknown 3 | NA | NA | 54 | 19.41 | 1357 | NA | ND | 4 | 2.7 | -0.59 | -1 | 6.3E-07 | 0.0 |
| MET_152 | 5-Hepten-2-one, 6-methyl- | 110-93-0 | HMDB0035915 | 73 | 19.42 | 1357 | 1338+-9 | 1351 | 1 | 3.5 | -0.83 | -1 | 6.0E-15 | 82.0 |
| MET_155 | Methylthio-2-propanone ^t^ | 14109-72-9 | HMDB0040170 | 60 | 19.5 | 1360 | NA | ND | 3 | 1.7 | -0.49 | -1 | 5.1E-05 | 81.9 |
| MET_175 | Dimethyl trisulfide | 3658-80-8 | HMDB0013780 | 79 | 20.85 | 1408 | 1377+-11 | 1405 | 1 | 1.1 | -0.35 | -1 | 4.6E-03 | 93.6 |
| MET_186 | Ethanol, 2-butoxy- | 111-76-2 | HMDB0031327 | 57 | 21.44 | 1430 | 1405+-12 | 1420 | 2 | 2.2 | 0.55 | 1 | 4.0E-07 | 91.8 |
| MET_190 | Unknown 4 | NA | NA | 57 | 21.5 | 1433 | NA | ND | 4 | 1.3 | 0.22 | 1 | 1.6E-02 | 0.0 |
| MET_195 | Unknown 5 | NA | NA | 43 | 21.76 | 1442 | NA | ND | 4 | 1.1 | 0.30 | 1 | 8.9E-03 | 0.0 |
| MET_197 | Unknown 6 | NA | NA | 73 | 21.82 | 1445 | NA | ND | 4 | 1.9 | -0.72 | -1 | 1.3E-07 | 0.0 |
| MET_199 | p-cymene ^t^ | NA | NA | 135 | 21.96 | 1450 | 1272+- 8 | 1278 | 3 | 1.0 | -0.30 | -1 | 1.6E-02 | 0.0 |
| MET_201 | Unknown 7 | NA | NA | 341 | 22.17 | 1458 | NA | ND | 4 | 2.1 | -0.68 | -1 | 2.8E-07 | 0.0 |
| MET_210 | 7-tetradecene, (E)- ^t^ | 41446-60-0 | HMDB0061833 | 55 | 22.66 | 1477 | 1420+-8 | ND | 3 | 1.9 | 0.49 | 1 | 1.2E-05 | 88.5 |
| MET_212 | Acetic acid | 64-19-7 | HMDB0000042 | 67 | 22.73 | 1479 | 1449+-13 | 1472 | 1 | 2.8 | 0.87 | 1 | 9.3E-20 | 96.9 |
| MET_215 | Unknown 8 | NA | NA | 59 | 22.89 | 1485 | NA | ND | 4 | 2.1 | 0.68 | 1 | 8.8E-12 | 0.0 |
| MET_216 | Unknown 9 | NA | NA | 68 | 22.93 | 1487 | NA | ND | 4 | 1.8 | 0.57 | 1 | 4.0E-07 | 0.0 |
| MET_217 | 2-propanone, 1-(acetyloxy)- | 592-20-1 | HMDB0034466 | 43 | 22.94 | 1487 | 1474+-9 | 1488 | 1 | 1.1 | 0.40 | 1 | 6.2E-04 | 94.0 |
| MET_222 | 2-pentanone, 4-hydroxy ^t^ | 4161-60-8 | NA | 43 | 22.98 | 1489 | NA | ND | 3 | 1.2 | 0.30 | 1 | 7.7E-03 | 92.4 |
| MET_224 | 1-hexanol, 2-ethyl- | 104-76-7 | HMDB0031231 | 57 | 23.33 | 1502 | 1491+-5 | 1499 | 1 | 1.6 | -0.41 | -1 | 1.4E-02 | 97.0 |
| MET_235 | Butanoic acid, 3-hydroxy-, methyl ester ^t^ | 1487-49-6 | HMDB0041603 | 43 | 23.64 | 1514 | 1461+-6 | ND | 3 | 2.2 | -0.60 | -1 | 2.3E-06 | 83.0 |
| MET_233 | Unknown 11 | NA | NA | 69 | 23.64 | 1514 | NA | ND | 4 | 2.2 | -0.55 | -1 | 6.2E-05 | 0.0 |
| MET_238 | Decanal | 112-31-2 | HMDB0011623 | 57 | 23.73 | 1518 | 1498+-8 | 1515 | 1 | 2.2 | -0.49 | -1 | 1.1E-02 | 97.8 |
| MET_247 | 1,2,3-butanetriol ^t^ | 4435-50-1 | HMDB0034778 | 103 | 23.96 | 1527 | NA | ND | 3 | 3.4 | -0.85 | -1 | 1.9E-13 | 69.5 |
| MET_248 | 2,5-hexanedione | 110-13-4 | HMDB0245506 | 43 | 24.11 | 1533 | 1500+-4 | 1537 | 1 | 1.8 | -0.53 | -1 | 5.3E-03 | 82.2 |
| MET_252 | 3-nonen-2-one | 14309-57-0 | HMDB0034764 | 55 | 24.19 | 1537 | 1515+-12 | 1539 | 1 | 2.5 | -0.64 | -1 | 9.9E-07 | 82.2 |
| MET_266 | Propanoic acid | 79-09-4 | HMDB0000272 | 74 | 24.79 | 1561 | 1535+-11 | 1570 | 1 | 1.7 | 0.37 | 1 | 3.7E-04 | 96.2 |
| MET_267 | Unknown 12 | NA | NA | 70 | 24.8 | 1561 | NA | ND | 4 | 1.6 | 0.40 | 1 | 2.8E-04 | 0.0 |
| MET_269 | cis-4-thujanol ^t^ | 15537-55-0 | HMDB0303503 | 71 | 24.97 | 1568 | 1465+-4 | ND | 3 | 1.6 | 0.46 | 1 | 5.5E-05 | 85.4 |
| MET_274 | Unknown 13 | NA | NA | 46 | 25.26 | 1580 | NA | ND | 4 | 2.0 | 0.45 | 1 | 8.3E-05 | 0.0 |
| MET_292 | Propanoic acid, 2,2-dimethyl- ^t^ | 75-98-9 | HMDB0041992 | 69 | 25.7 | 1597 | 1568+-6 | 1537 | 3 | 1.6 | -0.67 | -1 | 1.3E-07 | 93.1 |
| MET_320 | Terpinen-4-ol ^t^ | 562-74-3 | HMDB0035833 | 71 | 26.44 | 1628 | 1602+-9 | ND | 3 | 1.2 | 0.30 | 1 | 6.3E-03 | 95.3 |
| MET_368 | Levomenthol | 2216-51-5 | HMDB0003352 | 81 | 27.88 | 1690 | 1633+-19 | 1668 | 2 | 1.7 | 0.30 | 1 | 2.6E-02 | 84.3 |
| MET_392 | Unknown 14 | NA | NA | 117 | 28.69 | 1725 | NA | ND | 4 | 1.1 | -0.27 | -1 | 2.8E-02 | 0.0 |
| MET_398 | cis-verbenol ^t^ | 473-67-6 | HMDB0036129 | 109 | 28.74 | 1727 | 1674+-6 | ND | 3 | 1.2 | 0.35 | 1 | 8.5E-03 | 83.6 |
| MET_416 | Octane, 1,1'-oxybis- | 629-82-3 | HMDB0061856 | 43 | 29.4 | 1757 | 1761+-3 | 1754 | 1 | 4.6 | -1.11 | -1 | 2.1E-31 | 92.9 |
| MET_424 | Acetic acid, 2-methylphenyl ester ^t^ | 533-18-6 | HMDB0032073 | 107 | 29.51 | 1762 | 1716 | ND | 3 | 2.1 | 0.56 | 1 | 1.4E-07 | 95.6 |
| MET_437 | 1-cyclopentene-1-carboxylic acid ^t^ | 1560-11-8 | NA | 67 | 30.01 | 1784 | NA | 2145 | 3 | 1.2 | 0.25 | 1 | 4.2E-02 | 81.8 |
| MET_443 | Unknown 15 | NA | NA | 56 | 30.3 | 1797 | NA | ND | 4 | 1.1 | 0.31 | 1 | 4.7E-03 | 0.0 |
| MET_444 | Unknown 16 | NA | NA | 67 | 30.34 | 1799 | NA | ND | 4 | 1.7 | 0.56 | 1 | 3.2E-09 | 0.0 |
| MET_445 | Acetamide ^t^ | 60-35-5 | HMDB0031645 | 44 | 30.45 | 1804 | 1764+-11 | ND | 3 | 1.2 | 0.46 | 1 | 4.1E-05 | 98.2 |
| MET_447 | Unknown 17 | NA | NA | 99 | 30.57 | 1809 | NA | ND | 4 | 1.2 | 0.35 | 1 | 4.8E-04 | 0.0 |
| MET_456 | Ethanol, 1-(2-butoxyethoxy)- | 112-34-5 | HMDB0244918 | 57 | 30.99 | 1829 | 1796+-10 | 1822 | 1 | 1.7 | 0.62 | 1 | 5.1E-10 | 92.3 |
| MET_457 | Unknown 18 | NA | NA | 129 | 30.99 | 1829 | NA | ND | 4 | 1.9 | 0.64 | 1 | 1.5E-12 | 0.0 |
| MET_458 | Butanenitrile, 4-(methylthio)- | 59121-24-3 | HMDB0302370 | 61 | 31 | 1829 | 1770+-37 | 1835 | 1 | 1.7 | 0.59 | 1 | 6.0E-10 | 90.4 |
| MET_460 | Unknown 19 | NA | NA | 110 | 31.03 | 1831 | NA | ND | 4 | 2.0 | 0.64 | 1 | 6.4E-10 | 0.0 |
| MET_468 | 2H-pyran-2-one, tetrahydro-6-methyl- | 823-22-3 | HMDB0000453 | 70 | 31.39 | 1847 | 1791+-20 | 1855 | 1 | 1.0 | 0.37 | 1 | 1.7E-03 | 90.6 |
| MET_482 | Ethanol, 2-(2-butoxyethoxy)-, acetate ^t^ | 124-17-4 | NA | 43 | 31.87 | 1869 | 1838 | ND | 3 | 1.7 | 0.36 | 1 | 4.1E-03 | 90.4 |
| MET_544 | Phenylethyl alcohol | 60-12-8 | HMDB0033944 | 91 | 33.8 | 1962 | 1906+-15 | 1955 | 1 | 1.2 | 0.35 | 1 | 3.0E-04 | 96.3 |
| MET_546 | Unknown 20 | NA | NA | 43 | 33.81 | 1962 | NA | ND | 4 | 1.2 | 0.29 | 1 | 2.7E-03 | 0.0 |
| MET_554 | Pentanenitrile, 5-(methylthio)- ^t^ | 59121-25-4 | HMDB0302371 | 61 | 34.07 | 1975 | 1851+-1 | ND | 3 | 1.3 | 0.29 | 1 | 1.3E-02 | 84.5 |
| MET_563 | Creosol | 93-51-6 | HMDB0032136 | 138 | 34.44 | 1993 | 1956+-12 | 1998 | 1 | 1.0 | 0.33 | 1 | 2.3E-03 | 96.5 |
| MET_582 | Propane, 1-isothiocyanato-3-(methylthio)- | 505-79-3 | HMDB0034370 | 101 | 35.14 | 2028 | 1975+-5 | 2037 | 1 | 1.2 | -0.34 | -1 | 5.6E-03 | 93.4 |
| MET_588 | Isopropyl tetradecanoate | 110-27-0 | HMDB0040392 | 43 | 35.58 | 2050 | 2027+-7 | 2045 | 1 | 2.2 | -0.49 | -1 | 5.7E-06 | 97.6 |
| MET_600 | 1-phenoxypropan-2-ol | 770-35-4 | HMDB0341215 | 94 | 36.24 | 2083 | NA | 2084 | 1 | 1.7 | 0.37 | 1 | 1.1E-03 | 91.6 |
| MET_608 | Terpene | NA | NA | 81 | 36.64 | 2104 | NA | ND | 4 | 1.1 | -0.30 | -1 | 3.1E-02 | 0.0 |
| MET_613 | Unknown 21 | NA | NA | 57 | 36.88 | 2116 | NA | ND | 4 | 1.1 | -0.25 | -1 | 1.3E-02 | 0.0 |
| MET_614 | p-cresol | 106-44-5 | HMDB0001858 | 107 | 36.89 | 2117 | 2080+-12 | 2116 | 1 | 1.3 | 0.25 | 1 | 1.2E-02 | 98.4 |
| MET_617 | Unknown 22 | NA | NA | 57 | 36.94 | 2119 | NA | ND | 4 | 2.2 | -0.59 | -1 | 6.6E-09 | 0.0 |
| MET_635 | Nonanoic acid | 112-05-0 | HMDB0000847 | 60 | 38.26 | 2189 | 2171+-17 | 2184 | 1 | 1.7 | 0.36 | 1 | 1.7E-05 | 96.7 |
| MET_643 | 2-piperidinone | 675-20-7 | HMDB0011749 | 99 | 38.31 | 2192 | 2060 | 2214 | 2 | 1.3 | 0.33 | 1 | 6.9E-03 | 93.3 |
| MET_654 | Unknown 23 | NA | NA | 122 | 38.69 | 2212 | NA | ND | 4 | 1.9 | -0.37 | -1 | 3.3E-03 | 0.0 |
| MET_657 | 4-Isopropyl-2-methylphenol | 1740-97-2 | HMDB0038984 | 135 | 38.7 | 2213 | 2221 | ND | 2 | 1.8 | -0.33 | -1 | 4.6E-03 | 97.2 |
| MET_679 | 1H-pyrrole-2,5-dione, 3-ethyl-4-methyl- ^t^ | 20189-42-8 | NA | 124 | 40.49 | 2310 | 2244+-16 | ND | 3 | 1.3 | 0.30 | 1 | 1.0E-02 | 95.3 |
| MET_680 | Unknown 24 | NA | NA | 62 | 40.49 | 2310 | NA | ND | 4 | 1.4 | 0.30 | 1 | 2.3E-03 | 0.0 |
| MET_694 | Diethyl phthalate ^t^ | 84-66-2 | NA | 149 | 42.31 | 2414 | 2366+-8 | ND | 3 | 1.6 | -0.43 | -1 | 1.6E-03 | 97.9 |
| MET_701 | Nonanamide ^t^ | 1120-07-6 | NA | 59 | 45.37 | NA | NA | ND | 3 | 2.1 | -0.62 | -1 | 1.0E-09 | 79.1 |

The table lists 80 VOCs contributing to group separation in the OPLS-DA model. Columns include compound name, CAS number, HMDB ID (when available), mass-to-charge ratio (*m/z*), retention time, calculated and reference retention indices (RI), and RI from analytical standards. “VIP > 1” denotes variable importance in projection; “Direction” indicates relative abundance (1 = higher in cases, –1 = higher in controls). Statistical significance was evaluated by the Wilcoxon rank-sum test with FDR correction, and NIST spectral match scores (%) support compound annotation. Compounds marked “t” are tentatively identified. ND: not determined. NA: not available

**Supplementary Table S3** Complete KEGG/FELLA Output for Pathway Enrichment Analysis.

| **KEGG.id** | **Entry.type** | **KEGG.name** | **p.score** |
| --- | --- | --- | --- |
| hsa00640 | pathway | Propanoate metabolism - Homo sapiens (human) | 1e-6 |
| hsa04974 | pathway | Protein digestion and absorption - Homo sapiens | 1e-6 |
| hsa05034 | pathway | Alcoholism - Homo sapiens (human) | 1e-6 |
| 3.1.1.56 | enzyme | methylumbelliferyl-acetate deacetylase | 1e-6 |
| 3.1.2.1 | enzyme | acetyl-CoA hydrolase | 1e-6 |
| 3.4.11.9 | enzyme | Xaa-Pro aminopeptidase | 1e-6 |
| 3.4.16.2 | enzyme | lysosomal Pro-Xaa carboxypeptidase | 1e-6 |
| 3.4.17.15 | enzyme | carboxypeptidase A2 | 1e-6 |
| 3.4.23.1 | enzyme | pepsin A | 1e-6 |
| 3.4.24.18 | enzyme | meprin A | 1e-6 |
| 3.4.24.63 | enzyme | meprin B | 1e-6 |
| R00227 | reaction | acetyl-CoA hydrolase | 1e-6 |
| R00229 | reaction | acetate:CoA ligase (ADP-forming) | 1e-6 |
| R00326 | reaction | acetaldehyde:acceptor oxidoreductase | 1e-6 |
| R00710 | reaction | acetaldehyde:NAD+ oxidoreductase | 1e-6 |
| R00711 | reaction | acetaldehyde:NADP+ oxidoreductase | 1e-6 |
| R00746 | reaction | ethanol:NADP+ oxidoreductase | 1e-6 |
| R00754 | reaction | ethanol:NAD+ oxidoreductase | 1e-6 |
| R00920 | reaction | propanoate:CoA ligase (ADP-forming) | 1e-6 |
| R00925 | reaction | propanoate:CoA ligase (AMP-forming) | 1e-6 |
| R00928 | reaction | acetyl-CoA:propanoate CoA-transferase | 1e-6 |
| R01353 | reaction | ATP:propanoate phosphotransferase | 1e-6 |
| R01366 | reaction | acetoacetate carboxy-lyase (acetone-forming) | 1e-6 |
| R01550 | reaction | propan-2-ol:NADP+ oxidoreductase | 1e-6 |
| R01553 | reaction | acetone-cyanohydrin acetone-lyase (cyanide-forming) | 1e-6 |
| R02376 | reaction | propane-1,2-diol hydro-lyase | 1e-6 |
| R02377 | reaction | propane-1,3-diol:NAD+ 1-oxidoreductase | 1e-6 |
| R02611 | reaction | phenylethylalcohol:NAD+ oxidoreductase | 1e-6 |
| R02675 | reaction | 4-methylphenol:oxidized azurin oxidoreductase | 1e-6 |
| R05198 | reaction | ethanol:cytochrome c oxidoreductase | 1e-6 |
| R05298 | reaction | 4-hydroxy-2-oxohexanoate pyruvate-lyase | 1e-6 |
| R05666 | reaction | toluene,NADH:oxygen oxidoreductase | 1e-6 |
| R05917 | reaction | G00143 + H2O <=> G00144 + Acetate | 1e-6 |
| R07663 | reaction | Toluene-4-sulfonate + Oxygen <=> 4-Cresol + SO3 | 1e-6 |
| R09097 | reaction | propanal:NAD+ oxidoreductase (CoA-propanoylating) | 1e-6 |
| R09127 | reaction | ethanol:cytochrome c oxidoreductase | 1e-6 |
| R09479 | reaction | ethanol:quinone oxidoreductase | 1e-6 |
| R10246 | reaction | L-tyrosine 4-methylphenol-lyase | 1e-6 |
| R10703 | reaction | propan-2-ol:NAD+ oxidoreductase | 1e-6 |
| R10704 | reaction | acetone,NADPH:oxygen oxidoreductase | 1e-6 |
| R11773 | reaction | propionyl-CoA:succinate CoA transferase | 1e-6 |
| R13136 | reaction | propanoate:NAD+ oxidoreductase | 1e-6 |
| R13556 | reaction | Acrylic acid <=> Propanoate | 1e-6 |
| C00033 | compound | Acetate | 1e-6 |
| C00163 | compound | Propanoate | 1e-6 |
| C00207 | compound | Acetone | 1e-6 |
| C00469 | compound | Ethanol | 1e-6 |
| C00479 | compound | Propanal | 1e-6 |
| C01468 | compound | 4-Cresol | 1e-6 |
| C05853 | compound | Phenylethyl alcohol | 1e-6 |

This table reports the full output of the KEGG-based pathway analysis performed using the FELLA R package. For each KEGG entity identified (pathways, enzymes, reactions, and compounds), the table lists the corresponding KEGG identifier, entry type, KEGG name, and diffusion score (p.score). These results provide the detailed network context underlying the pathways highlighted in the main text.

# **References**

1.	Sun J, Xia Y. Pretreating and normalizing metabolomics data for statistical analysis. Genes Dis. 2024;11(3):100979. doi: 10.1016/j.gendis.2023.04.018.

2.	Tseng CY, Salguero JA, Breidenbach JD, Solomon E, Sanders CK, Harvey T, et al. Evaluation of normalization strategies for mass spectrometry-based multi-omics datasets. Metabolomics. 2025;21(4):98. doi: 10.1007/s11306-025-02297-1.

3.	Di Guida R, Engel J, Allwood JW, Weber RJ, Jones MR, Sommer U, Viant MR, Dunn WB. Non-targeted UHPLC-MS metabolomic data processing methods: a comparative investigation of normalisation, missing value imputation, transformation and scaling. Metabolomics. 2016;12:93. doi: 10.1007/s11306-016-1030-9.

4.	Antonelli J, Claggett BL, Henglin M, Kim A, Ovsak G, Kim N, et al. Statistical Workflow for Feature Selection in Human Metabolomics Data. Metabolites. 2019;9(7). doi: 10.3390/metabo9070143.

5.	Fuchsmann P, Tena Stern M, Munger LH, Pimentel G, Burton KJ, Vionnet N, Vergeres G. Nutrivolatilomics of Urinary and Plasma Samples to Identify Candidate Biomarkers after Cheese, Milk, and Soy-Based Drink Intake in Healthy Humans. J Proteome Res. 2020;19(10):4019-33. doi: 10.1021/acs.jproteome.0c00324.

6.	Sumner LW, Amberg A, Barrett D, Beale MH, Beger R, Daykin CA, et al. Proposed minimum reporting standards for chemical analysis Chemical Analysis Working Group (CAWG) Metabolomics Standards Initiative (MSI). Metabolomics. 2007;3(3):211-21. doi: 10.1007/s11306-007-0082-2.

7.	Viant MR, Kurland IJ, Jones MR, Dunn WB. How close are we to complete annotation of metabolomes? Curr Opin Chem Biol. 2017;36:64-9. doi: 10.1016/j.cbpa.2017.01.001.

8.	Tingley D, Yamamoto T, Hirose K, Keele L, Imai K. mediation: R Package for Causal Mediation Analysis. Journal of Statistical Software. 2014;59(5):1 - 38. doi: 10.18637/jss.v059.i05.

9.	Imai K, Keele L, Tingley D. A general approach to causal mediation analysis. Psychol Methods. 2010;15(4):309-34. doi: 10.1037/a0020761.
